# The pitfalls of “toughing it out”: mapping stoic attitudes in cancer patients. A scoping review

**DOI:** 10.1007/s11019-025-10293-4

**Published:** 2025-08-19

**Authors:** Alexis Harerimana, Julian David Pillay, Gugu Mchunu

**Affiliations:** 1 College of Healthcare Sciences, James Cook University, Townsville, Australia; 2 Faculty of Health Sciences, Durban University of Technology, Durban, South Africa

**Keywords:** Cancer patients, People with cancer, Health, Healthcare, Pseudo-stoicism, Stoic attitudes, Stoic ideology

## Abstract

Stoicism (with an upper-case ‘S’) as a life philosophy promotes resilience, self-control and rational acceptance of adversity. In contrast, lower-case stoicism, including pseudo-stoicism or stoic attitudes—characterised by emotional suppression and the silent endurance of pain or hardship—has been linked to adverse health outcomes among cancer patients. Thus, further research is needed to understand the drawbacks of stoic attitudes in cancer patients. This scoping review aims to map stoic attitudes in cancer patients, particularly in relation to potential health consequences. The review adhered to Levac et al.’s framework for scoping reviews. A systematic search was conducted from five electronic databases: CINAHL, Emcare, Medline Ovid, Scopus, and Web of Science. Manual searches were conducted using Google and Google Scholar. A total of 955 records were identified, 526 were screened (title and abstracts), and 450 were excluded. After reviewing 76 full-text articles, 12 studies satisfied the inclusion criteria for data extraction and thematic analysis, consisting of five qualitative and seven quantitative studies. A time frame of 10 years was considered, ranging from 2014 to 2024. This scoping review revealed that pseudo-stoic attitudes in cancer patients often lead to emotional suppression, reduced social support, delayed help-seeking and poor management of symptoms such as pain. These attitudes were linked to poorer psychological outcomes and underreporting of symptoms, especially among older males and rural cancer patients. Studies found that stoic traits were sometimes associated with persistence and treatment adherence among cancer patients. Pseudo-stoicism hinders emotional expression and delays help-seeking, leading to adverse health outcomes; however, stoic attitudes are also associated with adaptive qualities, such as psychological endurance and a commitment to care. Therefore, it is vital to promote balanced coping strategies that honour resilience while encouraging open emotional engagement among cancer patients.

## Background

Stoicism has been applied to health and illness behaviours in contemporary research (Pathak et al. 2017; Gomez et al. 2022; Martin 2023). Stoicism as a life philosophy, referred to as Stoicism with an upper-case ‘S’, emphasises alignment with nature and virtue cultivation (Isaacs 2023). Prominent Stoic philosophers include Zeno, Seneca, Epictetus, and Marcus Aurelius, who articulated essential principles such as controlling one’s responses and accepting fate (Isaacs 2023). Stoic philosophy promotes resilience, self-control, and rational decision-making amid adversity by encouraging individuals to focus on what they can control, thereby enhancing stress management and emotional well-being (Guerin 2022; Isaacs 2023; Dybwad and Pripp 2024). Brown et al. (2022) state that Stoic practices—including negative visualisation and mindfulness—enhance emotional preparedness and self-reflection. A minimalist interpretation of Stoicism may be particularly advantageous for contemporary practitioners navigating stress and change (Chakrapani 2022). Over time, the philosophy of Stoicism has been simplified, resulting in what is known as pseudo-stoicism, also referred to as lower-case ‘stoicism’ (Karl et al. 2022; McElheran et al. 2024). Lower-case stoicism or pseudo-stoicism (or stoic attitudes or naïve stoic ideology) frequently entails emotional suppression, resulting in detrimental psychological outcomes, such as increased stress and diminished well-being (Kelly 2021; Karl et al. 2022; McElheran et al. 2024). In this paper, the term ‘stoicism’ is used to denote lower-case stoicism, including stoic attitudes and pseudo-stoicism, except where explicitly stated otherwise.

The misinterpretation of Stoicism as a life philosophy diverges from its foundational aim of fostering inner strength and resilience through ethical conduct and rational thought (Limaj 2024). While people also promote pseudo-stoicism as a pathway to resilience and inner peace, it has faced criticism for potentially harming mental health. The focus on suppressing emotions and detachment can lead to psychological distress (Mah et al. 2017, 2018; Gomez et al. 2022). Furthermore, its links to toxic masculinity have contributed to the reinforcement of harmful gender norms (Táíwò 2020). Applying pseudo-stoicism to health behaviours is complex, with potential benefits and drawbacks, necessitating further research to fully understand its impact on health outcomes and patient care (Becker 2003; Spiers 2006; Corboy et al. 2019; Kaukiainen and Kolves 2020).

The stoic ideology (pseudo-stoicism) has been characterised by emotional non-expression and endurance, which correlates with negative well-being outcomes. While it may aid in pain coping, it can impede emotional processing and help-seeking behaviours (Karl et al. 2022). Furthermore, stoic ideology significantly influences the management of chronic conditions, affecting psychological and behavioural responses (Yong 2006; Fan et al. 2023). People who adopt a stoic ideology lack the willingness to disclose their symptoms, such as pain, to others (Turner 2021). While this may assist individuals in enduring symptoms, it may also contribute to underreporting and reluctance to seek medical care, complicating chronic condition management (Yong 2006). In COPD, stoic attitudes are linked to heightened anxiety and stress and correlate with reduced social support, yet they do not directly impact care-seeking behaviours (Fan et al. 2023). The management of chronic pain often involves stoic attitudes, which can lead to underreporting symptoms and thus hinder effective treatment and necessary adjustments (Jones et al. 2005; Mah et al. 2018). This underreporting often results from caution stemming from fears of disbelief or a desire to avoid burdening others (Jones et al. 2005; Spiers 2006; Yong 2006). Societal pressures to mask emotional or physical distress further complicate communication; Spiers (2006) notes that patients often suppress pain expressions to maintain dignity, adversely affecting relationships with healthcare providers and hindering effective treatment.

Given the perilous implications of cancer and the arduous nature of its treatment protocols, psychological turmoil is frequently observed among individuals diagnosed with cancer at every phase of the disease (Min et al. 2013; Ma et al. 2024). For patients with cancer, resilience is a crucial psychological attribute that enables individuals to navigate challenging situations while preserving mental and physical well-being (Min et al. 2013; Ma et al. 2024). Higher resilience correlates with enhanced problem-solving capabilities, increased creativity, and greater self-efficacy in managing challenges (Ma et al. 2024). While stoicism can enhance resilience and self-efficacy in specific contexts, it may also hinder cancer diagnosis and treatment by discouraging symptom expression and psychological help-seeking (Gomez et al. 2022). In cancer patients, stoicism is associated with lower perceived social support and optimism, leading to maladaptive coping strategies that adversely affect psychological well-being (Gomez et al. 2022). Furthermore, stoicism is particularly prevalent among older male cancer patients and is associated with depression, suggesting it may function more as a risk factor than a protective mechanism in pain management, leading to delayed help-seeking and pain underreporting (Castelo et al. 2018). Similarly, Gomez et al. (2022) found that in cancer patients, stoicism correlates with diminished social support, reduced optimism, and passive coping strategies.

The stoic ideology’s focus on self-reliance and emotional endurance can discourage seeking professional help for severe symptoms (Murray et al. 2008). Ultimately, comprehending the role of stoic ideology in chronic conditions is essential for devising interventions that meet patients’ psychological and social support requirements, ensuring stoic attitudes do not obstruct effective care and management (Mah et al. 2018; McAteer and Gillanders 2019; Gomez et al. 2022; Fan et al. 2023). Thus, this scoping review aims to map stoic attitudes in cancer patients, particularly in relation to potential health consequences.

## Methodology

This study followed a scoping review framework from Levac et al. (2010). The following steps from this framework were considered: identifying the research questions, identifying relevant studies, selecting the studies, charting the data, collating, summarising and reporting the findings.

### Identifying the research question

The scoping review process, as delineated by Levac et al. (2010), commenced with formulating a precise research question. The questions were designed to be specific enough to guide the review while remaining sufficiently broad to include diverse studies. The following research questions were considered in this study:

1. What are the tools used to measure stoic attitudes in cancer patients?
2. What are the effects of stoic attitudes on cancer patients’ health?
3. How do stoic attitudes relate to the sociodemographic profiles of cancer patients?

### Identifying relevant studies

Following the formulation of the research questions, a comprehensive search strategy was developed to identify pertinent studies, as Levac et al. (2010) recommended. This involved exploring multiple databases and additional sources, such as grey literature and manual searches. Databases included CINAHL, Emcare, Medline Ovid, Scopus, and Web of Science. Manual searches were conducted using Google and Google Scholar. A time frame of 10 years was considered, ranging from 2014 to 2024. The search terms were developed and adapted to each database. Below is an example of a search-term combination.

- S1: “Stoic Philosophy” OR “stoicism” OR “stoic” OR “stoic ideology” OR “stoic attitude” OR “stoic beliefs” OR “pseudo-stoicism”.
- S2: “Cancer patients” OR “oncology patients” OR “people with cancer” OR “people living with cancer”.
- S3: exp health/ OR exp health care/.
- S4: S1 AND S2 AND S3.

### Selecting relevant studies

Study selection followed a two-stage process, as Levac et al. (2010) suggested. Initially, titles and abstracts were screened against predefined criteria, followed by a full-text review to assess eligibility for inclusion. The studies included peer-reviewed articles and grey literature, published in English, such as dissertations that focused on the application of lower-case stoicism, pseudo-stoicism, or stoic attitudes in healthcare contexts involving cancer patients globally. Exclusion criteria comprised non-empirical studies, those not published in English and studies that did not address stoicism, pseudo-stoicism, or stoic attitudes within healthcare settings or among cancer patients. Additionally, the research questions and Mixed Methods Appraisal Tool (MMAT) guided the selection of the relevant studies. EndNote Version 20 was used to organise the sources.

**Fig. 1:**
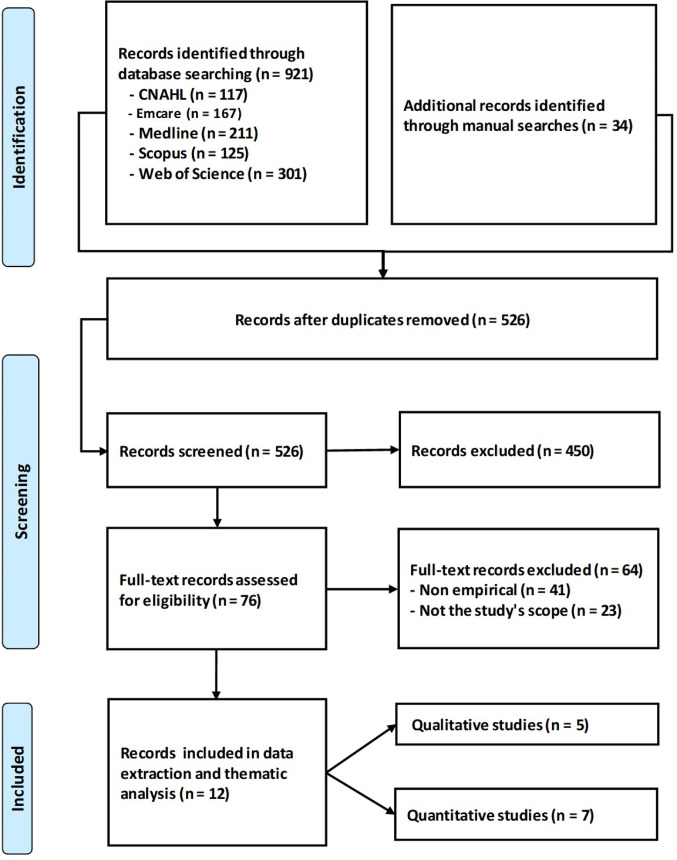
PRISMA flowchart

A total of 955 records were identified through both database and manual searches, which included 921 records obtained from five electronic databases—CINAHL (*n* = 117), Emcare (*n* = 167), Medline (*n* = 211), Scopus (*n* = 125), and Web of Science (*n* = 301)—alongside 34 records sourced from manual searches. After removing duplicates, 526 records proceeded to title and abstract screening, excluding 450 records. Subsequently, 76 full-text articles were evaluated for eligibility, of which 64 were excluded due to non-empirical content (*n* = 41) or irrelevance (*n* = 23). Ultimately, 12 studies satisfied the inclusion criteria for data extraction and thematic analysis, consisting of five qualitative and seven quantitative studies (Fig. 1).

### Charting the data

Data charting entailed systematically extracting relevant information from the included studies (Levac et al. 2010). A data-charting form was utilised to capture critical details, including author, year, study aims, study population, research designs and key results. The data-charting process was iteratively refined to enhance accuracy and ensure comprehensive information collection (Table 1). Furthermore, data related to the tools used in the measurements of the stoic attitudes were extracted and included the title of the tool/ questionnaire, abbreviated title, purpose of the tool, authors, country and reliability of the instruments per author (Cronbach's alpha) (Table 2).

**Table 1:** Summary of the findings

Authors	Country	Study title	Population Sample size	Research design	Main findings
Calderon et al. (2017)	Spain	“Psychometric properties of Liverpool Stoicism Scale (LSS) in a cohort of patients with resected cancer in adjuvant treatment”	Patients who had undergone surgery for non-advanced solid tumours and were candidates for adjuvant treatment (*n* = 443)	Quantitative	LSS exhibited acceptable psychometric properties, including a four-factor structure that provided a good fit to the data. Cancer patients in the study displayed moderate stoicism scores The personality trait of introversion was found to predict a portion of the variance in stoicism scores. The findings suggest that stoicism may influence clinical outcomes, such as treatment compliance and help-seeking behaviour
Corboy et al. (2019)	Australia	“Self-reliance and stoicism as predictors of distress following radical prostatectomy in the context of place of residence”	Australian men who had undergone radical prostatectomy (RP) within the previous six months (*n* = 447)	Quantitative	The research established self-reliance and stoicism as distinct predictors of psychological distress in men post-radical prostatectomy, controlling for sociodemographic and medical variables. The impact of residence on the stoicism-distress relationship was significant only for urban men, whereas self-reliance affected all men irrespective of location. Elevated stoicism correlated with higher psychological distress in urban men, a trend not observed in those from regional or remote areas. These results indicate that masculine values, especially self-reliance and stoicism, may exacerbate psychological distress after prostate cancer treatment, emphasising the necessity for tailored support interventions
Dobson et al. (2022)	United Kingdom	“Barriers to early presentation amongst rural residents experiencing symptoms of colorectal cancer: a qualitative interview study”	Participants with symptoms of colorectal cancer (*n* = 720, with 40 selected for interviews)	Qualitative	A desire to rule out cancer as the cause of symptoms was a key motivator for early presentation among rural residents. Stoicism and self-reliance were barriers to early presentation, as rural residents tended to manage symptoms independently and only consult when symptoms became unmanageable. Time constraints, particularly for self-employed individuals, were a barrier to early presentation, as the time lost from work to consult a doctor was seen as a significant burden. The quality of the patient-GP relationship was a key factor in willingness to consult, with poor relationships being a barrier, particularly for “native” rural residents
Gomez et al. (2022)	Spain	“Stoic attitude in patients with cancer from the NEOcoping study: Cross-sectional study”	Patients with non-metastatic, resected cancer (*n* = 932)	Quantitative	The study revealed a negative correlation between stoicism in cancer patients and perceived social support, optimism, and positive attitudes, suggesting that increased stoicism corresponds with decreased supportive factors. In males, stoicism showed a positive correlation with maladaptive coping strategies such as helplessness and anxious preoccupation, alongside anxiety and depression. Women demonstrated analogous trends, with stoicism positively correlated with age and maladaptive coping strategies, while negatively associated with perceived social support and optimism. The study indicated that patients with elevated stoicism scores experienced greater psychological distress, including depression, in comparison to those with lower scores. The findings suggest that while stoicism may offer specific coping mechanisms, it could also impede the expression of distress and the pursuit of medical assistance, potentially resulting in adverse outcomes
MacLean et al. (2017)	United Kingdom	“Does gender matter? An analysis of men’s and women’s accounts of responding to symptoms of lung cancer”	Patients diagnosed with lung cancer (*n* = 45)	Qualitative	There were more similarities than differences in how men and women accounted for their interpretations of and responses to symptoms of lung cancer. Both men and women presented themselves as stoic in response to symptoms and responsible health service users, and they portrayed themselves as having sought explanations for their symptoms and taken sensible, appropriate and timely actions. No significant differences existed in how men and women presented their moral identities or justified their use of health services
Mah et al. (2017)	Canada	“Psychometric evaluation of the pain attitudes questionnaire-revised for people with advanced cancer”	Patients with advanced cancer (*n* = 269)	Quantitative	Stoicism was multidimensional, encompassing fortitude (enduring pain), concealment (hiding pain), and superiority (belief in higher pain tolerance), and was measured using the PAQ-R. Older adults did not report higher stoicism than younger adults, contradicting the common belief that stoicism increases with age. Younger adults showed more stoic superiority, indicating they believed they could tolerate or control pain better than others. Fortitude and superiority were linked to lower pain intensity in older adults, suggesting potential protective effects of some stoic traits in this group. In younger adults, higher concealment and fortitude were associated with higher pain intensity and interference, implying these attitudes may worsen pain outcomes when pain is unexpressed
Mah et al. (2018)	Canada	“Do correlates of pain-related stoicism and cautiousness differ in younger and older people with advanced cancer?”	Outpatients with advanced cancer and cancer-related pain (*n* = 269)	Quantitative	Age differences in pain attitudes were absent in advanced cancer patients, with both younger and older showing similar stoicism and cautiousness. Younger patients faced relational issues, while older patients dealt with intrapersonal fears related to avoidance. Medical correlates varied by age, with younger patients focusing on symptoms and older patients on ageing-related issues. Activity engagement correlated for both groups, but younger patients had more distinct psychosocial correlates. The study emphasised a biopsychosocial approach to pain adaptation, advocating for age-specific considerations in pain management
McAteer and Gillanders (2019)	United Kingdom	“Investigating the role of psychological flexibility, masculine self-esteem and stoicism as predictors of psychological distress and quality of life in men living with prostate cancer”	Men diagnosed with prostate cancer (*n* = 311)	Quantitative	Psychological flexibility strongly predicted psychological distress and quality of life in men with prostate cancer. Prostate cancer symptoms also significantly influenced distress and quality of life. The study showed no significant correlation between stoicism and psychological outcomes in this group. This indicated that stoic attitudes might not significantly impact psychological outcomes for these men. Masculine self-esteem was suggested as a treatment focus, advocating interventions to foster healthier masculine identities. Age significantly affected outcomes, with younger men experiencing lower quality of life and older men reporting higher psychological distress
Pulgar et al. (2015)	Spain	“Psychosocial predictors of quality of life in haematological cancer”	Hematological cancer patients (*n* = 69)	Quantitative	Stoicism was linked to a decreased ability to fulfil physical roles and poor emotional function. In the first regression model, physical function negatively affected passivity, accounting for 16% of the variance. When depression was included in the second model, both variables explained 22% of the variance. Additionally, the physical role was negatively associated with the coping strategy of stoicism, explaining 10% of the variance. In the first model, the emotional role was associated with stoicism, explaining 12% of the variance; when the strategy of relaxation was included in the second model, the two variables together accounted for 19% of the variance
Staneva et al. (2018)	Australia	“The imperative for a triumph-over-tragedy story in women’s accounts of undergoing chemotherapy for ovarian cancer”	Women who completed chemotherapy for cancer (*n* = 18)	Qualitative	Women with ovarian cancer felt isolated and different from women with other types of cancer due to a lack of formal peer support groups specific to ovarian cancer. To cope with this isolation, women adopted a stoic and strong identity, presenting a positive and triumphant narrative about their chemotherapy experience, even when it involved significant pain and suffering. The imperative to present a positive, triumphant narrative of surviving cancer treatment masked the underlying difficulties and distress that many women experienced, which they were hesitant to express openly
Staneva et al. (2019)	Australia	“[I Wasn’t Gonna Let It Stop Me]: Exploring women’s experiences of getting through chemotherapy for ovarian cancer”	Women who completed chemotherapy for cancer (*n* = 18)	Qualitative	Women with ovarian cancer attributed various factors as helpful during chemotherapy, including positioning themselves as stoic and strong, engaging in self-care activities, and relying on a strong support system. The “stoic woman” identity represented women’s active and optimistic attitude during their treatment, which was often reinforced by others like their doctors. Women engaged in specific self-care activities during treatment, such as “nourishing their body while poisoning the cancer” through diet, exercise, and alternative therapies. Emotional and practical support from family and healthcare providers was vital for women to complete their treatment
Whitaker et al. (2015)	United Kingdom	“Help-seeking for cancer ‘alarm’ symptoms: a qualitative interview study of primary care patients in the UK”	Adults aged 50 years and older with some cancer alarming symptoms (*n* = 48)	Qualitative	Reasons for seeking help included symptom persistence, social influence, awareness/fear of a link with cancer, and ‘just instinct’. Reasons for not seeking help included belief that the symptom was trivial/normalising symptoms, stoicism, fear, worry about wasting the doctor’s time, and lack of confidence in the healthcare system. Negative attitudes towards interactions with the GP were widespread, with people worried about wasting the doctor’s time, reporting difficulty making appointments and a lack of continuity of care. A Significant role of stoicism, with participants expressing a desire to “just tolerate” symptoms and distance themselves from being seen as someone who “goes running to the doctor”, as this was viewed as a sign of weakness

**Table 2:** Standardised instruments for measuring stoic attitudes and associated psychological, physical, social, spiritual and Health-Related outcomes in cancer patients

Abbreviation	Tool/ Questionnaire title	Purpose	Authors	Countries	Reliability (α)
LSS	Liverpool Stoicism Scale	Measures stoic attitudes	Calderon et al. (2017)	Spain	0.83
			Corboy et al. (2019)	Australia	0.81
			Gomez et al. (2022)	Spain	0.83
PWSIS	Pathak–Wieten Stoicism Ideology Scale	Assesses stoic beliefs within the context of an illness	McAteer and Gillanders (2019)	United Kingdom	0.82
BFI-10	Big Five Inventory-10	Assesses personality traits	Calderon et al. (2017)	Spain	0.75 to 0.90
PAQ-R	Pain Attitudes Questionnaire-Revised	Measures pain-related stoicism (fortitude, concealment, superiority) and cautiousness (self-doubt, reluctance) in chronic pain	Mah et al. (2018)	Canada	0.72 to 0.87
			Mah et al. (2017)	Canada	0.73 to 0.80
LOT-R	Revised Life Orientation Test	Assesses optimism and life orientation	Gomez et al. (2022)	Spain	0.74 to 0.78
LOT	Life Orientation Test	Assesses life orientation and optimism	Pulgar et al. (2015)	Spain	0.87
BSI-18	Brief Symptom Inventory-18	Measures psychological distress	Corboy et al. (2019)	Australia	0.94
			Gomez et al. (2022)	Spain	0.81 to 0.90
HAD	Hospital Anxiety and Depression Scale	Assesses anxiety and depression	Pulgar et al. (2015)	Spain	0.82 to 0.84
DASS-21	Depression Anxiety Stress Scale-21	Evaluates emotional states like depression, anxiety, and stress	McAteer and Gillanders (2019)	United Kingdom	0.95
CES-D	Centre for Epidemiologic Studies-Depression Scale	Measures depressive symptoms	Mah et al. (2018)	Canada	0.88 to 0.89
FACT-G	Functional Assessment of Cancer Therapy-General	Assesses well-being in cancer patients	McAteer and Gillanders (2019)	United Kingdom	0.92
FACT-PCS	Functional Assessment of Cancer Therapy-Prostate Cancer Subscale	Measures well-being specific to prostate cancer	Corboy et al. (2019)	Australia	0.78
			McAteer and Gillanders (2019)	United Kingdom	0.79
Mini-MAC	Mini-Mental Adjustment to Cancer	Assesses coping strategies in cancer patients	Gomez et al. (2022)	Spain	0.52 to 0.90
ISEAC	Stressors and Coping Strategies for Cancer Inventory	Explores cancer-related stressors and coping mechanisms	Pulgar et al. (2015)	Spain	0.80
IES	Impact of Event Scale	Measures emotional responses to trauma	Mah et al. (2018)	Canada	0.79 to 0.85
FACIT-Sp-12	Functional Assessment of Chronic Illness Therapy-Spiritual Well-being-12	Evaluates spiritual well-being in illness contexts	Mah et al. (2018)	Canada	0.87 to 0.90
CCI	Charlson Comorbidity Index	Assesses the presence of comorbidities	Mah et al. (2018)	Canada	Not reported (NR)
SOMCT	Short Orientation Memory Concentration Test	Measure’s cognitive function	Mah et al. (2018)	Canada	NR
BPI	Brief Pain Inventory	Measures severity of pain and impact on daily life	Mah et al. (2017)	Canada	NR
			Mah et al. (2018)	Canada	NR
ESAS	Edmonton Symptom Assessment System	Evaluates symptom severity in cancer patients	Mah et al. (2018)	Canada	0.86 to 0.88
CPAQ	Chronic Pain Acceptance Questionnaire	Measures acceptance of chronic pain	Mah et al. (2018)	Canada	0.81 to 0.88
PCS	Pain Catastrophising Scale	Assesses negative thought patterns related to pain	Mah et al. (2018)	Canada	0.75 to 0.90
PASS-20	Pain Anxiety Symptom Scale-20	Measures fear and anxiety associated with pain	Mah et al. (2018)	Canada	0.69 to 0.87
MSPSS	Multidimensional Scale of Perceived Social Support	Assesses perceived social support	Gomez et al. (2022)	Spain	0.90
SF-36	Short-Form Health Survey	Measures overall health and well-being	Pulgar et al. (2015)	Spain	0.70 to 0.94
MOS-SS	Medical Outcomes Study Social Support Survey	Assesses perceived social support	Mah et al. (2018)	Canada	0.88 to 0.94
KMS	Kansas Marital Satisfaction Scale	Measures marital satisfaction	Mah et al. (2018)	Canada	0.98
AS-25	Social Support Scale	Evaluates social support	Pulgar et al. (2015)	Spain	0.87
ECR	Experiences in Close Relationships Inventory	Assesses interpersonal attachment styles	Mah et al. (2018)	Canada	0.89 to 0.91
MPI-C	Multidimensional Pain Inventory Caregivers Responses Scale	Evaluates caregiver responses to patient pain	Mah et al. (2018)	Canada	0.73 to 0.81
NCS-BHSS	Need for Control and Self-Reliance Subscale of the Barriers to Help Seeking Scale	Measures barriers to seeking help related to control/self-reliance	Corboy et al. (2019)	Australia	0.83
MSES	Masculine Self-Esteem Scale	Assesses male self-esteem/ masculinity following the diagnosis and or treatment of prostatic cancer	McAteer and Gillanders (2019)	United Kingdom	0.91
CompACT	Comprehensive Assessment of Acceptance and Commitment Therapy	Measures psychological flexibility	McAteer and Gillanders (2019)	United Kingdom	0.89

### Collating, summarising, and reporting the results

The results were collated, summarised, and reported using quantitative and qualitative methodologies. A thematic analysis was conducted on the key findings guided by the research questions. Quantitative and qualitative data were integrated to offer a thorough comprehension. The PRISMA-ScR guidelines were used to systematically and transparently document and report the findings of the scoping review.

## Results

### Studies’ characteristics

Twelve studies were included in this scoping review—exhibiting diverse research environments and methodologies across four countries: four studies from the United Kingdom (Whitaker et al. 2015; MacLean et al. 2017; McAteer and Gillanders 2019; Dobson et al. 2022), three studies were from Spain (Pulgar et al. 2015; Calderon et al. 2017; Gomez et al. 2022), three studies from Australia (Staneva et al. 2018, 2019; Corboy et al. 2019), and two from Canada (Mah et al. 2017, 2018). Methodologically, seven studies (58.3%) utilised quantitative approaches to analyse extensive patient data, emphasising specific treatments and clinical outcomes using standardised measurement tools (Pulgar et al. 2015; Calderon et al. 2017; Mah et al. 2017, 2018; Corboy et al. 2019; McAteer and Gillanders 2019; Gomez et al. 2022). Conversely, five studies (41.7%) applied qualitative methods to explore patient experiences and perceptions (Whitaker et al. 2015; MacLean et al. 2017; Staneva et al. 2018, 2019; Dobson et al. 2022). Sample sizes varied significantly, with qualitative studies ranging from 18 participants (Staneva et al. 2018, 2019) to a quantitative study encompassing 932 participants (Gomez et al. 2022). A total of 3589 participants were involved in the 12 reviewed studies. Each study was counted individually, even if some participants might have been included in more than one study by the same authors. In this paper, unless otherwise specified, the term “stoicism” denotes lower-case stoicism or stoic attitudes or pseudo-stoicism.

### Measurement tools of stoic attitudes in cancer patients

A total of 33 measurement tools were utilised collectively across seven quantitative studies. Several of these studies employed multiple scales to assess stoic attitudes, often combining instruments that specifically referenced stoicism—such as the Liverpool Stoicism Scale (LSS)—with others that measured related constructs, including emotional suppression, coping styles, psychological distress, and pain perception. This multi-scale approach underscores the multidimensional nature of stoic attitudes and highlights the importance of using complementary measures to fully capture their impact on psychological, emotional, and physical outcomes. Furthermore, the application of multiple tools across these studies demonstrates a robust methodology for understanding the multifactorial nature of patient experiences in cancer contexts.

Table 2 presents the various measurement tools used in different studies to comprehensively assess stoic attitudes and associated psychological, physical, social, spiritual and health-related outcomes in cancer patients. For instance, Mah et al. (2018) utilised 15 tools, the highest measurement diversity, while McAteer and Gillanders (2019) employed six instruments to evaluate stoicism, psychological distress, and quality of life. Furthermore, Corboy et al. (2019) applied four tools to investigate psychological outcomes and help-seeking barriers. Two studies, Gomez et al. (2022) and Pulgar et al. (2015), used five instruments, focusing on stoic attitudes, quality of life, and coping mechanisms. A study by Mah et al. (2017) incorporated three tools related to pain attitudes and symptom evaluation, while Calderon et al. (2017) used two instruments to assess personality and stoicism. The internal consistency coefficients of the measurement tools ranged from α = 0.52 to 0.98 (Table 2), demonstrating adequate to excellent reliability across health contexts (Pulgar et al. 2015; Calderon et al. 2017; Mah et al. 2017, 2018; Corboy et al. 2019; McAteer and Gillanders 2019; Gomez et al. 2022).

Stoic attitude or stoicism was measured using the Liverpool Stoicism Scale (LSS) (Calderon et al. 2017; Corboy et al. 2019; Gomez et al. 2022) and the Pathak–Wieten Stoicism Ideology Scale (PWSIS) (McAteer and Gillanders 2019). Personality traits were assessed through the Big Five Inventory 10 (BFI 10) (Calderon et al. 2017). Pain-related stoicism and chronic pain attitudes were evaluated with the Pain Attitudes Questionnaire-Revised (PAQ-R) (Mah et al. 2017, 2018), while dispositional optimism was measured using the Life Orientation Test (LOT) (Pulgar et al. 2015) and its revised version (LOT-R) (Gomez et al. 2022). Physical symptom severity and pain interference were evaluated with the Edmonton Symptom Assessment System (ESAS) (Mah et al. 2018) and Brief Pain Inventory (BPI) (Mah et al. 2017, 2018), supplemented by measures of pain acceptance (CPAQ) (Mah et al. 2018), catastrophising (Pain Catastrophising Scale) (Mah et al. 2018), and pain-related anxiety (Pain Anxiety Symptom Scale 20 (Mah et al. 2018).

Emotional distress, encompassing anxiety, depression, and stress, was assessed via the Brief Symptom Inventory 18 (BSI 18) (Corboy et al. 2019; Gomez et al. 2022), Hospital Anxiety and Depression Scale (HAD) (Pulgar et al. 2015), Depression Anxiety Stress Scale 21 (DASS 21) (McAteer and Gillanders 2019) and the Centre for Epidemiologic Studies Depression Scale (CES-D) (Mah et al. 2018). Cancer-specific quality of life and adjustment were measured using the Functional Assessment of Cancer Therapy General (FACT G) and its prostate-specific subscale (FACT PCS) (Corboy et al. 2019; McAteer and Gillanders 2019). Coping responses were evaluated with the Mini-Mental Adjustment to Cancer (Mini MAC) (Gomez et al. 2022) and the Impact of Event Scale (IES) (Mah et al. 2018). Spiritual well-being was assessed using the FACIT Sp 12 (Mah et al. 2018), comorbidity burden via the Charlson Comorbidity Index (CCI) (Mah et al. 2018), and cognitive function with the Short Orientation Memory Concentration Test (SOMCT) (Mah et al. 2018).

Social support and interpersonal dynamics were assessed using the Multidimensional Scale of Perceived Social Support (MSPSS) (Gomez et al. 2022), Medical Outcomes Study Social Support Survey (MOS SS) (Mah et al. 2018), Social Support Scale (AS 25) (Pulgar et al. 2015), Kansas Marital Satisfaction Scale (KMS) (Mah et al. 2018), Experiences in Close Relationships Inventory (ECR) (Mah et al. 2018), and the Multidimensional Pain Inventory Caregiver Responses Scale (MPI-C) (Mah et al. 2018). Barriers to help-seeking were evaluated with the Need for Control and Self-Reliance Subscale (NCS-BHSS) (Corboy et al. 2019), masculine self-esteem through the Masculine Self-Esteem Scale (MSES) (McAteer and Gillanders 2019), and psychological flexibility via the Comprehensive Assessment of Acceptance and Commitment Therapy (CompACT) (McAteer and Gillanders 2019). Qualitative studies from the United Kingdom and Australia have employed structured interview guides to investigate themes related to stoicism (Whitaker et al. 2015; MacLean et al. 2017; Staneva et al. 2018, 2019; Dobson et al. 2022). The use of diverse measurement tools—both those with and without “stoicism” in their names—enables a comprehensive exploration of stoicism and its relationship with psychological and health-related outcomes. These instruments allow researchers to assess explicit stoic traits as well as related constructs such as emotional regulation, distress, and coping strategies. By combining these tools, studies achieve a more nuanced and valid understanding of how stoic traits manifest in various contexts, and the multifaceted challenges encountered by cancer patients.

### Effects of stoic attitudes in cancer patients

#### Help-seeking and stoic attitudes

Most studies indicated that stoicism (pseudostoicism) negatively affects the timely pursuit of medical help, while a smaller number found that it can positively influence patients’ adherence to treatment plans. For instance, six studies (Whitaker et al. 2015; MacLean et al. 2017; Staneva et al. 2018; Corboy et al. 2019; McAteer and Gillanders 2019; Dobson et al. 2022) revealed that pseudo-stoicism often leads to delays in seeking medical attention for cancer symptoms. People with stoic attitudes tend to downplay their symptoms, viewing them as minor or manageable without professional help. Furthermore, two studies identified that stoic attitudes contributed to delays in cancer therapy and psychological support, particularly among elderly patients (Calderon et al. 2017; Mah et al. 2018). A study by Whitaker et al. (2015) found that older adults often resist seeking medical assistance as they believe it may “waste the doctor’s time.” Similarly, a study by Calderon et al. (2017) identified a positive association between pseudo-stoicism and age, indicating that stoic ideology was observed in older patients.

A study by Dobson et al. (2022) revealed that in rural communities, cultural norms promoting stoic attitudes discouraged individuals from reporting symptoms early, even when they might signal serious health issues. Additionally, Corboy et al. (2019) found that stoic traits among urban men recovering from prostate cancer surgery made them less likely to seek psychological support, which increased their levels of distress. Thus, stoic attitudes served as a barrier to showing vulnerability or seeking mental health resources (Corboy et al. 2019). MacLean et al. (2017) observed that both men and women frequently justified their inaction by portraying their stoic identity as being a “good” or “responsible” patient, thus avoiding the impression of being overly concerned or burdensome.

#### Pain management and stoic attitudes

Stoic attitudes significantly affect how cancer patients perceive, report, and manage pain. Two psychometric studies utilising the Pain Attitudes Questionnaire-Revised (PAQ-R) identified concealment—a reluctance to disclose pain—as a critical stoic dimension associated with under-reporting (Mah et al. 2017, 2018). Younger patients exhibiting high concealment scores reported greater pain intensities, leading to delayed analgesic adjustments and increased pain-related interference in daily functioning (Mah et al. 2018). Fortitude, another PAQ-R subscale reflecting the ability to endure pain without complaint, was more prevalent among older adults. In two studies (Mah et al. 2017, 2018), elevated fortitude scores correlated with lower perceived average pain intensity, suggesting that stoic endurance may influence pain perception. However, these patients often requested fewer analgesics, heightening the risk of under-treated pain. A related study by Pulgar et al. (2015) indicated that stoic coping was associated with greater physical role limitations on the SF-36 quality of life measure in hematologic cancer patients, underscoring the functional consequences of unacknowledged pain.

Three quantitative studies (Calderon et al. 2017; Corboy et al. 2019; Gomez et al. 2022) linked stoicism to reluctance in engaging with pain management services. Calderon et al. (2017), using the Liverpool Stoicism Scale, demonstrated that patients with high stoicism scores delayed accessing palliative care or pain specialists, opting instead to “tough it out.” Gomez et al. (2022) found a negative correlation between stoicism and perceived social support—more stoic individuals had fewer interpersonal advocates for pain relief. Additionally, Corboy et al. (2019) indicated that self-reliant men post-prostatectomy reported lower engagement with pain medications despite heightened distress.

Despite its drawbacks, four studies (Calderon et al. 2017; Mah et al. 2018; Staneva et al. 2018, 2019) indicated that stoicism might support treatment perseverance, thus promoting psychological resilience and patients adhering to rigorous treatment protocols. Staneva et al. (2019) found that stoic attitudes enabled women with ovarian cancer to complete chemotherapy despite significant pain. Mah et al. (2018) also found that fortitude positively correlated with continued participation in life activities, suggesting that certain stoic traits enhance functional coping. The evidence from this review indicates that stoicism has a dual impact on symptom management. Studies of stoicism in the context of cancer adaptation demonstrate that high levels of stoic attitudes were detrimental (Corboy et al. 2019; Gomez et al. 2022). Pseudo-stoicism has been associated with lower perceived social support, decreased optimism, and a preference for passive coping strategies, all of which hinder adaptation to cancer (Gomez et al. 2022).

#### Emotional suppression and its consequences

Emotional suppression was identified across 12 studies analysed, frequently linked to stoicism and identified as a coping mechanism among cancer patients to uphold strength, autonomy, or social identity; while it occasionally fostered resilience, it more often hindered emotional expression, delayed symptom disclosure, compromised mental health and poor quality of life. Seven studies highlighted emotional suppression as a core aspect of stoicism (pseudo-stoicism) (Pulgar et al. 2015; Calderon et al. 2017; Mah et al. 2017, 2018; Staneva et al. 2018, 2019; Gomez et al. 2022). Mah et al. (2017) and Mah et al. (2018), utilising the Pain Attitudes Questionnaire-Revised (PAQ-R), identified concealment as a quantifiable trait of emotional suppression. Patients with high concealment scores were more likely to suppress both physical and emotional pain expressions, resulting in underreporting and suboptimal pain management outcomes. Gomez et al. (2022), through the Liverpool Stoicism Scale, found suppression strongly correlated with depression, helplessness, and anxiety, while inversely related to optimism and social support. A study by Calderon et al. (2017) indicated that the statement related to emotional concealment—“I tend not to express my emotions”—explained 20% of the variance.

Six qualitative studies (Whitaker et al. 2015; MacLean et al. 2017; Staneva et al. 2018, 2019; Corboy et al. 2019; Dobson et al. 2022) characterised emotional suppression as a socially reinforced behaviour influenced by cultural, gendered, and moral expectations. In the context of ovarian cancer, Staneva et al. (2018) and Staneva et al. (2019) reported that women felt compelled to construct positive, stoic narratives during chemotherapy, concealing their suffering to maintain a “strong” identity. MacLean et al. (2017) similarly observed that both men and women practised emotional restraint to present themselves as morally responsible patients, avoiding burdening healthcare systems.

Five studies connected emotional suppression to gendered expressions of masculinity, particularly among men with prostate or lung cancer (Calderon et al. 2017; MacLean et al. 2017; Corboy et al. 2019; McAteer and Gillanders 2019; Gomez et al. 2022). Corboy et al. (2019) found that men adhering to stoic masculinity were less inclined to seek psychological support and more likely to internalise distress. McAteer and Gillanders (2019) reported that stoic suppression did not alleviate distress but contributed to a lower emotional quality of life. Emotional suppression also served as a barrier to communication with healthcare providers in four studies (Whitaker et al. 2015; Calderon et al. 2017; Dobson et al. 2022; Gomez et al. 2022). Patients frequently withheld emotional concerns due to fear of judgment or appearing weak, limiting opportunities for timely psychosocial intervention.

The stoic emotional suppression is consistently linked to increased emotional distress—anxiety, depression, and stress among cancer patients. A study by Corboy et al. (2019) reported that high levels of emotional suppression may lead to poor mental health outcomes, particularly among men with prostate cancer who may avoid discussing their emotional experiences. In the context of cancer adaptation, research also suggested that elevated levels of stoic attitudes can have harmful effects (Gomez et al. 2022). Stoic attitudes have been associated with lower perceived social support, decreased optimism, and a preference for passive coping strategies, all of which hinder adaptation to cancer (Gomez et al. 2022). A study by Mah et al. (2018) discovered that patients who scored high on emotional concealment were more likely to report symptoms of depression. Similarly, Gomez et al. (2022) found that higher scores on the Liverpool Stoicism Scale were associated with increased anxiety and depression levels, as well as stress responses in cancer patients.

In addition to quantitative studies, four qualitative studies have shown that suppressing emotions contributes to persistent psychological distress (Whitaker et al. 2015; MacLean et al. 2017; Staneva et al. 2018, 2019). Whitaker et al. (2015) reported that older adults often downplayed persistent symptoms and avoided seeking help—often associated with the fear that their symptoms might be related to cancer and investigations.

### Stoic attitudes and sociodemographic profiles of cancer patients

Seven studies linked stoic ideals to age, gender norms and masculinity (Calderon et al. 2017; MacLean et al. 2017; Mah et al. 2017, 2018; Corboy et al. 2019; McAteer and Gillanders 2019; Gomez et al. 2022). A study by Gomez et al. (2022) explored how stoic attitude relates to psychological factors and coping strategies in 932 patients with non-metastatic cancer, and there were notable differences between genders—men showed significantly higher levels of stoic attitudes than women, who experienced more physical symptoms, anxiety, and depression. For men, higher stoicism was linked to lower social support and optimism and increased anxiety (Gomez et al. 2022). In women, higher stoic attitudes were associated with older age, less social support, and greater feelings of helplessness (Gomez et al. 2022). Men also perceived a greater risk of cancer recurrence without chemotherapy and underestimated the toxicity risks of chemotherapy compared to women (Gomez et al. 2022). Additionally, older patients and those with colon cancer, who were mostly men, displayed higher stoic attitudes than younger patients and those with breast cancer, who were primarily women (Gomez et al. 2022).

A study by Corboy et al. (2019) found that higher levels of stoic attitudes were linked to increased psychological distress in men living in urban areas. Stoic attitudes among men diagnosed with prostate cancer have been found to significantly affect their psychological adjustment to the illness, with notable implications for their overall quality of life (McAteer and Gillanders 2019). Dobson et al. (2022) found that entrenched cultural valorisation of self-sufficiency resulted in delayed help-seeking for colorectal symptoms, with vulnerability perceived as socially unacceptable. MacLean et al. (2017) demonstrated that both genders adopted stoic identities to align with the cultural norms of a responsible patient who avoids burdening others.

Evidence from four studies indicated that the social bias linked to the stoic trait, characterised by enduring pain without outward expression, disproportionately affected specific populations (Whitaker et al. 2015; Mah et al. 2017, 2018; Dobson et al. 2022). Studies by Mah et al. (2017) and Mah et al. (2018) found that older adults perceived as resilient report lower pain levels; however, they encounter unaddressed challenges in daily functioning, highlighting how societal norms regarding pain management can increase vulnerability. Furthermore, Mah et al. (2018) explored the role of age in stoic responses to chronic conditions, finding that younger cancer patients exhibited symptom-focused stoic attitudes while older patients demonstrated age-related stoic attitudes. Similarly, Calderon et al. (2017) identified that old age was correlated with higher stoicism scores on the Liverpool Stoicism Scale.

Two studies, Dobson et al. (2022) and Whitaker et al. (2015), demonstrated that rural patients and older individuals face compounded social pressures, balancing expectations to maintain stoic traits while contending with limited healthcare access, leading to heightened isolation and care delays. Emotional suppression fosters a cycle of isolation, and societal expectations can exacerbate this issue, compelling individuals to minimise emotions (Calderon et al. 2017). MacLean et al. (2017) similarly noted that patients feared judgment for appearing emotionally vulnerable. Societal expectations of perpetual positivity can foster self-blame and self-directed social judgment, compelling patients to mask their vulnerability (Pulgar et al. 2015; Staneva et al. 2018, 2019). Demographic factors such as age, gender, and place of residence reinforce normative pressures to appear stoic, often culminating in silent and unaddressed patient distress.

## Discussion

Studies analysed in this scoping review provided robust evidence regarding the influence of stoic attitudes on cancer patients. These influences manifest in various dimensions, including patients’ willingness to seek assistance, pain management, emotional expression, and overall well-being (Pulgar et al. 2015; Whitaker et al. 2015; Calderon et al. 2017; MacLean et al. 2017; Mah et al. 2017, 2018; Staneva et al. 2018, 2019; Corboy et al. 2019; McAteer and Gillanders 2019; Dobson et al. 2022; Gomez et al. 2022).

A significant finding indicates that stoic attitudes among cancer patients can serve as a barrier to seeking timely medical assistance. Patients who scored high on standardised measurement tools to assess stoic attitudes and health-related constructs in cancer patients (see Table 2) tended to minimise or conceal their symptoms, often perceiving medical consultations as unnecessary or burdensome to healthcare providers (Moore et al. 2013; Whitaker et al. 2015; MacLean et al. 2017; Pathak et al. 2017). The literature shows that individuals with chronic conditions or disabilities often conceal their health needs to avoid being perceived as weak or overly dependent (Page et al. 2020; Shadrina 2024). This tendency is particularly pronounced among older adults and individuals residing in rural areas, where a stoic attitude is frequently regarded as a moral virtue (Moore et al. 2013; Calderon et al. 2017; Dobson et al. 2022). Similarly, Purc-Stephenson et al. (2024) reported that in rural communities, cultural norms around stoicism and fear of vulnerability discourage help-seeking, further perpetuating silent suffering. Furthermore, the literature shows that the delays in seeking assistance can considerably impact the prognosis and treatment outcomes for cancer patients (Pathak et al. 2017).

Furthermore, stoicism influenced patients’ perceptions and management of pain. Research employing the PAQ-R demonstrated that characteristics such as concealment and fortitude, integral to stoicism, were associated with underreporting of pain and reduced pain medication utilisation (Mah et al. 2017, 2018). Older patients tended to endure pain without complaint, while younger patients who concealed their pain reported heightened discomfort and greater disruption to their daily lives. This suggests that, although stoicism may be perceived as a form of resilience, it could also lead to delays and avoidance of help-seeking, inadequate symptom management and a diminished quality of life (Pulgar et al. 2015; Pathak et al. 2017; Gomez et al. 2022). Similarly, Lane and Smith (2018) reported that older generations, conditioned to endure pain, often underreport or avoid seeking help, viewing pain as a natural aspect of ageing or a manifestation of moral strength, which complicates accurate pain assessment.

Emotional suppression emerged as a prevalent theme associated with stoic ideology, as numerous patients expressed the need to conceal their emotional struggles to project strength or conform to societal expectations regarding gender (MacLean et al. 2017; Staneva et al. 2018; Corboy et al. 2019). This phenomenon was particularly pronounced among men, who frequently equated emotional expression with weakness (Corboy et al. 2019; Gomez et al. 2022). However, emotional suppression can precipitate adverse outcomes, including heightened anxiety, depression, and stress (Mah et al. 2018; Gomez et al. 2022), and may impede patients’ access to necessary psychosocial support (Whitaker et al. 2015; Dobson et al. 2022). The literature shows that anxiety and depression are the most frequently experienced psychological symptoms in cancer patients, regardless of the stage of the disease, the type of cancer, or the treatment phase they are undergoing (Die Trill 2013; Goerling et al. 2023; Grassi et al. 2023). Emotion regulation is an important mediator of resilience in cancer patients (Vaughan et al. 2019). Interventions aimed at enhancing resilience should be accessible to cancer patients who are interested and motivated to participate (Ludolph et al. 2019). These support programmes should be offered alongside medical treatments right from the moment of diagnosis (Ludolph et al. 2019).

Variables such as age, gender, and cultural background influenced the expression of stoicism and its effects on patients. For instance, older men tended to display greater stoicism and diminished emotional expressiveness, often leading to increased psychological distress (Gomez et al. 2022). Women also exhibited stoic traits but frequently did so to foster a positive atmosphere during treatment, potentially obscuring their actual need for support (Staneva et al. 2019). Older women often avoided seeking help out of fear of appearing dependent, prioritising others’ well-being to fit cultural expectations of caregiving and self-sufficiency (Shadrina 2024). These gender disparities reflect broader societal norms that valorise self-reliance and emotional restraint, particularly in health crises.

Interestingly, some studies indicated that stoic attitudes could serve as an effective coping mechanism. For example, Mah et al. (2018) and Staneva et al. (2019) observed that certain patients demonstrated perseverance and continued treatment despite physical and emotional challenges. This “optimistic tenacity” facilitated their functional capacity and adherence to demanding treatment regimens. However, these beneficial effects were not universally consistent; in several studies, stoic traits were correlated with poorer mental health, increased distress, and reduced social support (McAteer and Gillanders 2019; Gomez et al. 2022), underscoring the necessity of differentiating between adaptive coping strategies and detrimental emotional suppression. This should not be interpreted as suggesting that Stoicism (with an upper-case ‘S’), the philosophical tradition, leads to such negative outcomes; in fact, evidence points to its beneficial effects (Brown et al. 2022). Furthermore, despite its downsides, lower-case stoicism provides useful insights when applied thoughtfully (McCarthy et al. 2021; Brown et al. 2022). Stoic principles, integrated into therapeutic practices, promote emotional balance and resilience, encouraging individuals to navigate stress with rationality and virtue (Brown et al. 2022; Hajizade et al. 2024; Schimmels et al. 2024). This perspective can help reduce psychological distress, improve life satisfaction, and foster personal growth when paired with strategies that support emotional expression and mental health awareness (Dickinson 2024; Schimmels et al. 2024).

### Implications of the study

This study underscores the necessity of recognising stoicism as a coping mechanism that can sometimes obscure emotional distress and impede appropriate care. Healthcare providers should employ validated assessment tools like the LSS, PWSIS and PAQ-R to identify patients who may minimise their symptoms or emotional needs. Early detection of elevated levels of emotional suppression can facilitate timely referrals to specialists in psycho-oncology and pain management. Communication strategies must be adapted to account for gender and cultural norms that valorise stoic endurance. Framing seeking assistance as a demonstration of strength rather than weakness may contribute to diminishing stigma, particularly among older adults, men, and individuals residing in rural areas. Educational initiatives targeting patients and caregivers can challenge detrimental beliefs and promote open dialogues regarding pain and emotional health. Given that stoicism can yield beneficial and adverse effects—encouraging resilience in some individuals while exacerbating suffering in others—oncology teams must differentiate between advantageous traits (such as optimistic determination) and detrimental emotional suppression. Regular psychosocial assessments should be integrated into treatment and survivorship plans to deliver tailored support. Future research should adopt a long-term, mixed-methods approach to examine how stoic attitudes evolve throughout the cancer experience and how they intersect with various demographic factors. On a broader scale, training for healthcare professionals should emphasise identifying stoic attitudes, applying culturally sensitive communication, and creating environments that foster emotional expression and the pursuit of assistance.

### Strengths and limitations

This scoping review thoroughly synthesises evidence on stoicism in cancer care, integrating quantitative and qualitative findings across various settings. A significant strength is its identification of validated measurement tools and exploration of stoicism’s multidimensional effects on help-seeking behaviour, pain management, and emotional well-being. Sociodemographic and cultural factors such as age, gender, and rurality enhance the understanding of patient behaviours. The study offers valuable insights for refining psychosocial assessments and patient-centred cancer care interventions by addressing both the adaptive and maladaptive aspects of stoicism.

However, this review presents some limitations. The studies included were from high-income countries, and the geographical distribution was notably limited, lacking representation from low- and middle-income contexts. This limitation hinders the generalizability of the findings to diverse cultural and healthcare environments, where the conceptualisation and expression of stoicism may differ significantly.

Additionally, the studies included in the review used different designs, populations, and methods for measuring stoicism. This variety highlights the complexity of stoicism and limits the ability to reach consistent conclusions about its psychological and behavioural impacts on different patient groups. Moreover, most quantitative studies were cross-sectional, limiting the ability to establish causality or observe changes over time. Although qualitative studies offered essential insights, they relied on self-reported data, which could be biased since people with stoic traits might downplay or underreport their emotional experiences.

## Conclusion

This scoping review revealed that stoic attitudes significantly affect cancer patients’ illness experiences, symptom management, and psychological well-being. Pseudo-stoic behaviours—such as emotional suppression, self-reliance, distress concealment, and fortitude—were consistently linked to delayed help-seeking, under-reporting of symptoms, and limited psychosocial support engagement. While stoicism was sometimes associated with persistence and treatment adherence in the present review, its predominant effect was detrimental to mental health outcomes. Patients engaging in emotional suppression reported heightened anxiety, depression, and stress, particularly among those conforming to societal or gendered expectations of strength, especially men and rural patients. Emotional suppression, a critical feature of stoic ideology, emerged as a maladaptive coping mechanism. Several studies demonstrated that patients adopted “pseudo-stoicism,” a performative stoicism aimed at fulfilling social expectations or preserving identity, masking significant emotional suffering. Although some individuals derived strength from their stoic identities, the overarching evidence suggests that rigid stoicism hampers emotional processing, exacerbates isolation, and undermines holistic recovery. These findings underscore the need to recognise stoicism in clinical contexts not as a neutral trait, but as a potential barrier to emotional disclosure and mental health care access.

Psycho-oncology services must foster an environment that encourages vulnerability and the expression of emotion without stigma and promote balanced coping strategies that honour resilience while facilitating open emotional engagement. Psychosocial assessments are crucial in creating personalised treatment and survivorship plans for cancer patients to ensure that individuals receive the appropriate support they need throughout their journey. Future research should adopt a long-term, mixed-methods approach to explore how stoic attitudes change during the cancer experience and how these attitudes relate to various demographic factors. It is also vital to train healthcare professionals to recognise stoic attitudes, communicate in a culturally sensitive manner, and create environments that promote emotional expression and encourage patients to seek help.
